# The CDC Domestic Mpox Response — United States, 2022–2023

**DOI:** 10.15585/mmwr.mm7220a2

**Published:** 2023-05-19

**Authors:** Jennifer H. McQuiston, Christopher R. Braden, Michael D. Bowen, Andrea M. McCollum, Robert McDonald, Neal Carnes, Rosalind J. Carter, Athalia Christie, Jeffrey B. Doty, Sascha Ellington, S. Nicole Fehrenbach, Adi V. Gundlapalli, Christina L. Hutson, Rachel E. Kachur, Aaron Maitland, Christine M. Pearson, Joseph Prejean, Laura A. S. Quilter, Agam K. Rao, Yon Yu, Jonathan Mermin

**Affiliations:** ^1^Division of High Consequence Pathogens and Pathology, National Center for Emerging Zoonotic Infectious Diseases, CDC; ^2^Office of the Director, National Center for Emerging Zoonotic Infectious Diseases, CDC; ^3^Division of STD Prevention, National Center for Immunization and Respiratory Diseases, CDC; ^4^Division of HIV Prevention, National Center for HIV, Viral Hepatitis, STD, and TB Prevention, CDC; ^5^Office of the Director, National Center for Immunization and Respiratory Diseases, CDC; ^6^Office of the Director, Global Health Center, CDC; ^7^Influenza Division, National Center for Immunization and Respiratory Diseases, CDC; ^8^Division of Preparedness and Emerging Infections, National Center for Emerging Zoonotic Infectious Diseases, CDC; ^9^Office of the Director, Public Health Science and Surveillance, CDC; ^10^Division of Health Interview Statistics, National Center for Health Statistics, CDC; ^11^Office of the Director, National Center for HIV, Viral Hepatitis, STD, and TB Prevention, CDC.

Monkeypox (mpox) is a serious viral zoonosis endemic in west and central Africa. An unprecedented global outbreak was first detected in May 2022. CDC activated its emergency outbreak response on May 23, 2022, and the outbreak was declared a Public Health Emergency of International Concern on July 23, 2022, by the World Health Organization (WHO),* and a U.S. Public Health Emergency on August 4, 2022, by the U.S. Department of Health and Human Services.^†^ A U.S. government response was initiated, and CDC coordinated activities with the White House, the U.S. Department of Health and Human Services, and many other federal, state, and local partners. CDC quickly adapted surveillance systems, diagnostic tests, vaccines, therapeutics, grants, and communication systems originally developed for U.S. smallpox preparedness and other infectious diseases to fit the unique needs of the outbreak. In 1 year, more than 30,000 U.S. mpox cases were reported, more than 140,000 specimens were tested, >1.2 million doses of vaccine were administered, and more than 6,900 patients were treated with tecovirimat, an antiviral medication with activity against orthopoxviruses such as *Variola virus *and *Monkeypox virus*. Non-Hispanic Black (Black) and Hispanic or Latino (Hispanic) persons represented 33% and 31% of mpox cases, respectively; 87% of 42 fatal cases occurred in Black persons. Sexual contact among gay, bisexual, and other men who have sex with men (MSM) was rapidly identified as the primary risk for infection, resulting in profound changes in our scientific understanding of mpox clinical presentation, pathogenesis, and transmission dynamics. This report provides an overview of the first year of the response to the U.S. mpox outbreak by CDC, reviews lessons learned to improve response and future readiness, and previews continued mpox response and prevention activities as local viral transmission continues in multiple U.S. jurisdictions (Figure).

**FIGURE:**
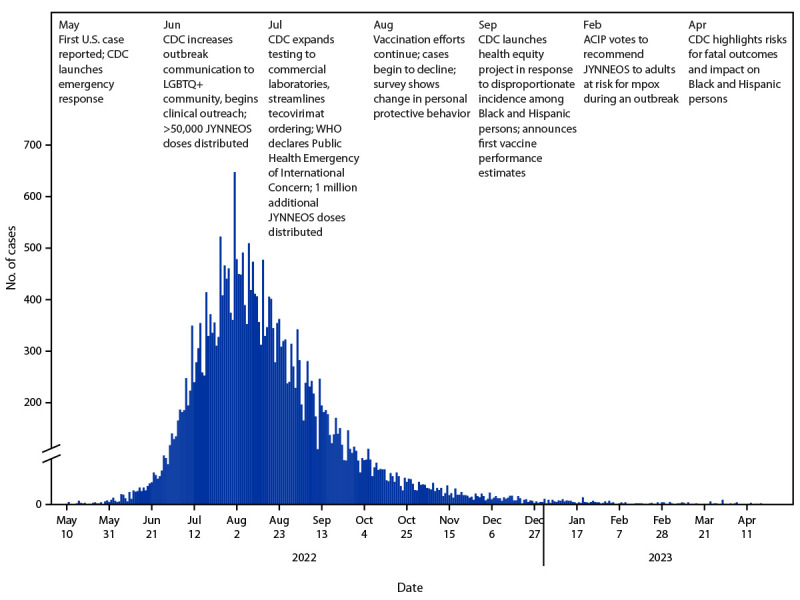
Mpox outbreak and CDC response — United States, May 2022–April 2023 **Abbreviations:** ACIP = Advisory Committee on Immunization Practices; LGBTQ+ = lesbian, gay, bisexual, transgender, queer, and other; mpox = monkeypox; WHO = World Health Organization.

## Epidemiology and Clinical Management of Cases

CDC partnered with state and local health departments to identify cases, analyze trends, and implement public health measures. Initial cases of mpox were associated with international travel, and cases associated with domestic transmission were quickly identified throughout all 50 states, the District of Columbia, and Puerto Rico. Case numbers peaked in early August 2022; as of May 10, 2023, a total of 30,395 cases and 42 mpox-associated deaths were reported to CDC. An overwhelming majority of cases occurred among adult MSM and persons aged 21–55 years (Table). The outbreak disproportionately affected persons with HIV: 38% of cases occurred among persons with known HIV. Health disparities were observed among racial and ethnic minority groups, including Black (33%) and Hispanic persons (31%). These disparities have been more pronounced among fatal cases: among 38 mpox-associated deaths reported through March 7, 2023, Black persons accounted for 33 (87%). Among decedents with information available, 94% were immunocompromised because of HIV infection (*1*). CDC investigations found that in addition to male-to-male sexual contact, transmission also occurred, albeit uncommonly, through close household contact (including from a caregiver), heterosexual sexual contact, and injury from a sharp object (e.g., needles and scalpels) (*2*–*5*). Three cases occurred in infants born to mothers who reported peripartum mpox symptoms.

**TABLE:** Demographic characteristics of persons with outbreak-associated mpox (N = 29,988)* — United States, May 2022–April 12, 2023

Characteristic (no. with available data)	No. of cases (%)
**Gender (29,988)**	
Cisgender man	28,535 (95)
Cisgender woman	878 (3)
Transgender man	67 (<1)
Transgender woman	273 (1)
Other gender	235 (1)
**Age group, yrs (29,988)**	
≤10	45 (<1)
11–15	16 (<1)
16–20	678 (2)
21–55	27,936 (93)
>55	1,313 (4)
**Race and ethnicity (28,350)^†^**	
American Indian or Alaska Native, non-Hispanic	124 (<1)
Asian, non-Hispanic	805 (3)
Black or African American, non-Hispanic	9,359 (33)
Native Hawaiian or other Pacific Islander, non-Hispanic	78 (<1)
White, non-Hispanic	8,373 (30)
Hispanic or Latino	8,798 (31)
Multiple races or other	813 (3)

* https://www.cdc.gov/poxvirus/mpox/response/2022/demographics.html (Accessed April 21, 2023).

**^†^** Data are missing for 1,638 cases.

The clinical presentation of cases in this outbreak (caused by clade IIb) was often different from those in historic reports. Whereas disseminated skin lesions had been previously considered a hallmark of mpox (*6*), many cases in the current outbreak involved small, localized skin lesions, with some patients experiencing symptoms of proctitis. Before this outbreak, tecovirimat had only been administered for rare orthopoxvirus infections, and apart from animal studies, safety and effectiveness data were lacking. During the outbreak, however, tecovirimat was widely used under an expanded access, investigational new drug (IND) protocol.^§^ As of April 25, 2023, tecovirimat had been administered to 6,932 patients.

In September 2022, clinical consultants at CDC began to receive queries about treatment of patients who were severely ill with mpox, including persons with disseminated infections attributed to uncontrolled viral replication due to severe immunocompromise, particularly in persons with advanced HIV disease (*7*). Early optimization of immune status recovery with the administration of antiretroviral therapy was identified as important to improving patient outcomes.

## Data-Driven Public Health Response

As part of national smallpox preparedness, CDC had previously developed a Food and Drug Administration (FDA)–approved nonvariola orthopoxvirus (NVO) polymerase chain reaction test^¶^ for use at Laboratory Response Network (LRN) laboratories in 68 U.S. locations; in May 2022, the capacity was 6,000 tests per week. By early July 2022, in collaboration with other government partners including FDA, NVO testing capacity was expanded to five commercial laboratory companies with broad U.S. coverage, thereby increasing national testing capacity to more than 80,000 specimens per week. As of April 6, 2023, a total of 144,209 NVO tests had been performed.

CDC successfully leveraged a SARS-CoV-2 wastewater surveillance system for mpox, with 533 testing sites across the country. During July 6, 2022–April 16, 2023, a total of 20,928 wastewater samples were collected; results from 162 sites (30%) were positive at any time, and positive results were often associated with geographic areas where cases had been reported. Although numbers of mpox cases in the United States have continued to decline, continued testing remains useful as an early warning signal; during April 2023, only 1% of sites reported positive test results.

CDC deposited viral sequence data from the first U.S. case specimen into a public database** within 3 days of diagnosis. To date, CDC has analyzed more than 7,000 sequences within databases and deposited 366 whole genomes into public databases, thereby helping to monitor viral strains and potential tecovirimat resistant mutations; fewer than 1.0% of sequences examined had mutations associated with in vitro resistance. When such mutations were identified, CDC attempted viral isolation and cell culture–based testing to confirm resistance, and most resistant specimens were associated with severely immunocompromised patients on prolonged courses of tecovirimat treatment. The collection of sequence data, resistance testing results, and clinical outcomes will help guide future decisions about tecovirimat use and any regulatory actions by FDA.

JYNNEOS, a third-generation smallpox vaccine approved for both smallpox and mpox, was used widely during the response. Because of limited supplies during May–June 2022, CDC initially recommended that vaccine be prioritized for postexposure prophylaxis of known contacts; this was later expanded to include persons at potential risk for recent exposure. Decisions on equitable distribution of vaccine were made by the U.S. government and included consideration of the number of mpox cases per state and estimates of at-risk populations. Aided by FDA’s August 9, 2022, authorization for a dose-sparing intradermal administration strategy in adults, 1.2 million doses of JYNNEOS were administered during May 2022–May 2023, resulting in 37% first-dose coverage of the estimated at-risk population and 23% completed second-dose vaccination coverage, nationally. Rapidly conducted vaccine effectiveness (VE) analyses using data reported from state and local health officials demonstrated that mpox incidence among unvaccinated persons was 9.6 times higher than that among persons who received 2 doses of vaccine (*8*). A matched case control study found an adjusted VE of 85.9% for 2 doses and 75.2% for 1 dose across all routes of administration, with a lower VE of 70.2% among fully vaccinated immunocompromised persons (*9*); no new or unexpected safety concerns were identified, and serious adverse events were rare (*10*). Outbreak data were instrumental in the Advisory Committee on Immunization Practices’ 2023 vote^††^ in favor of the use of JYNNEOS for adults at risk for acquiring mpox during an outbreak. The course of the outbreak during the ensuing months and years will help guide future vaccine administration policies.

## Clinician and Community Engagement and Communications

To familiarize U.S. health care professionals with mpox, CDC released seven Health Alert Network notices (https://emergency.cdc.gov/han) and held six Clinician Outreach and Communication Activity calls, beginning in May 2022, to provide clinicians with up-to-date information, including images of the different stages of mpox rash on various skin tones, and to communicate about nontraditional rash locations including anal-genital and oropharyngeal sites. Regular updates to clinical care recommendations provided rapid dissemination of advancing knowledge of diagnostics and vaccine and therapeutics use.

A health equity approach guided CDC’s response; an effort to ensure that the voices of affected communities were heard and honored was central to response activities. CDC tested messages and participated in approximately 50 community engagement and listening sessions to develop and refine mpox-related communications. Listening sessions included 26 owners and operators of sex-on-premises venues, 16 sex workers, and 28 harm reduction organizations. Early dissemination of key messaging via dating apps and lesbian, gay, bisexual, transgender, queer, and other (LGBTQ+) media targeted communities disproportionately affected by mpox. CDC resources were adopted and promoted by some prominent LGBTQ+ advocates and influencers, amplifying prevention messages. By August 2022, a survey of MSM indicated that 50% of respondents reported a reduction in the numbers of sex partners, one-time sexual encounters, and sex with partners met on dating apps or at sex-on-premises venues, which coincided with a significant decrease in reported mpox cases (*11*). Effectively communicating prevention messages to at-risk populations, including the importance of vaccination, is a critical component of continued prevention efforts.

Recognizing racial and ethnic disparities in incidence, illness outcomes, and vaccination coverage, CDC partnered with community organizations in 22 locations to pilot innovative vaccine equity projects to improve vaccination among disproportionately affected populations, including administering >25,000 vaccine doses at various locations across the nation, such as the largest HIV-related conference in the United States, at large festivals and Pride events, and through mobile vans (*12*,*13*).

## Lessons Learned and Future Challenges

Understanding the trajectory and subsequent mitigation of the outbreak was dependent upon collection and analysis of timely and reliable data on mpox cases, laboratory testing, and administration of countermeasures, such as vaccine and treatments that were tracked by state and local partners and health care providers and reported to CDC. Multiple successes were recognized, as were learning moments that can be studied and leveraged, which could lead to a more robust response to future epidemics. Control of the mpox outbreak was dependent on years of research related to diagnostics, therapeutics and vaccine development and approval, surveillance systems enhancements, and community partnerships. This type of research and preparation needs to continue for smallpox, mpox, and other biothreats. Public health agencies at all levels and the affected communities have made substantial progress in the year since the outbreak began, but the outbreak is not over. The United States remains at risk for increasing mpox transmission and reignited outbreaks. Continued prevention efforts are needed to interrupt viral transmission in countries where the virus is not endemic and to control transmission in areas were zoonotic mpox is endemic.

Based on decades of U.S. smallpox research, an FDA-approved test and surveillance network, as well as approved vaccines and therapeutics, were already in place and available to be used for mpox. Limited early availability of JYNNEOS vaccine required careful allocation to facilitate receipt of vaccine by persons at highest risk for exposure, with eligibility criteria expanding as additional vaccine supplies became available. This outbreak underscored the need to anticipate higher demand for JYNNEOS in the event of a future smallpox or mpox outbreak, as well as for advance planning for equitable distribution of and access to limited or scarce resources. The 2022–2023 mpox outbreak was driven by sexual contact as the most common mode of transmission, primarily among MSM populations. High rates of HIV in the affected population, as well as risks for significant side effects associated with ACAM2000 (a second generation smallpox vaccine) drove interest in JYNNEOS during this outbreak. The mpox outbreak response highlighted that early reporting requirements for the tecovirimat IND were too cumbersome for practical use during an outbreak; accordingly, CDC worked to simplify IND reporting requirements, reducing barriers to therapeutic administration.

Substantial LRN laboratory capacity was in place at the start of the mpox outbreak; however, easier access to and higher capacity for testing through commercial platforms was requested by providers. CDC worked quickly with other federal partners and commercial laboratories to rapidly increase national testing capacity and access within 2 months. These government and commercial partnerships were crucial during the height of the outbreak; similar partnerships are important to consider in future outbreak preparedness and response planning.

Because of the atypical clinical presentation and transmission dynamics in this outbreak compared with historic reports of mpox, rapid sharing of clear information was essential, and CDC’s ability to rapidly develop and disseminate messages and tools for partners during the mpox response hinged on leveraging expertise from sexual health and HIV prevention programs. Early engagement with and respect for persons at risk for mpox were central to CDC’s communications strategy with a focus on cultural sensitivity and competency (*14*). Rapid epidemiologic analyses and incorporation of findings into changing messages for risk reduction were also critical. However, even with these efforts, early messaging might have missed opportunities to improve understanding of sexual transmission risks among MSM. Communications evolved with the outbreak, with bidirectional feedback from communities and community champions critical to the learning process. CDC also relied on frequent communications with federal officials, state and local health officials, and WHO to ensure timely sharing of new information and joint prioritization.

During the outbreak, persons from racial and ethnic minority groups accounted for a large number of cases and severe outcomes, and a low proportion of persons receiving vaccines. Concerted efforts to reach affected communities with vaccination events mitigated but did not overcome these disparities. Equitable access to prevention and care, a critical component of strategy and policy, is ideally addressed before an outbreak but is also critical during the response. Assuring continued access to vaccines for persons at increased risk for mpox, increasing second-dose coverage, and closing equity gaps remain important goals to reduce the risk for mpox resurgence in the United States and worldwide, including having an ample supply of vaccine in countries in Africa with endemic disease to rapidly respond to outbreaks.

Research to evaluate the effectiveness, safety, and immunogenicity of JYNNEOS for mpox prevention is ongoing, with a need to determine whether booster immunization is needed. New medical countermeasures are being investigated, as well as continued molecular surveillance of the virus to monitor for mutations that might affect efficacy of current therapeutics. Studies at the animal-human interface for mpox continue, including the identification of animal reservoirs in countries that have historically had endemic mpox. New diagnostic assays, including specific detection of antibodies to the virus causing mpox in humans and animals, are in development.

The elimination of human transmission is a near-term goal for many countries where mpox is not endemic.^§§^ The association of mpox cases with HIV infection highlights the need for a syndemic approach to care for HIV, sexually transmitted infections, and mpox in the context of comprehensive sexual health care. The mpox outbreak occurred with little warning, peaked quickly, and waned 5 months after the first case was reported in the United States. Even as WHO declared the outbreak no longer a public health emergency on May 11, 2023, a cluster of mpox cases occurred in Chicago, Illinois, including among some previously vaccinated persons, demonstrating the ongoing risk for new cases and outbreaks and the need for continued vigilance and prevention efforts.^¶¶^ CDC continues to focus on surveillance, vaccination, and communication for populations at risk for mpox as important prevention tools.

**Summary**

**What is already known about this topic?**

After being detected in May 2022, U.S. monkeypox (mpox) cases increased rapidly, peaking in August. Infection was primarily spread by sexual contact among gay, bisexual, and other men who have sex with men.

**What is added by this report?**

Rapid adaptation of smallpox preparedness systems and tools, and prioritized communication expertise from HIV prevention programs, were leveraged to reach communities at risk. In 1 year, more than 30,000 cases were reported and >1 million JYNNEOS vaccine doses were administered. Black and Hispanic persons represented 33% and 31% of cases, respectively; 87% of 42 fatal cases occurred in Black persons.

**What are the implications for public health practice?**

The U.S. risk for future mpox outbreaks remains. Ongoing surveillance, vaccination, and communication are important prevention tools, especially for Black and Hispanic persons in groups at risk.

## Footnotes

*
https://www.who.int/europe/news/item/23-07-2022-who-director-general-declares-the-ongoing-monkeypox-outbreak-a-public-health-event-of-international-concern

†
https://www.hhs.gov/about/news/2022/08/04/biden-harris-administration-bolsters-monkeypox-response-hhs-secretary-becerra-declares-public-health-emergency.html

§
https://www.cdc.gov/poxvirus/mpox/clinicians/Tecovirimat.html

¶
https://www.cdc.gov/poxvirus/mpox/pdf/non-variola-orthopoxvirus-generic-real-time-pcr-test.pdf

**
https://ncbiinsights.ncbi.nlm.nih.gov/2022/05/26/monkeypox-virus-genome/

††
https://www.cdc.gov/vaccines/acip/recommendations.html

§§
https://www.who.int/europe/publications/i/item/WHO-EURO-2023-6007-45772-69163#

¶¶
https://www.chicagohan.org/alert-detail/-/alert-details/46678186
